# *GLUT1* rs1385129G>A Raised the Risk and Poor Prognosis of Lung Cancer: A Case‐Control Study

**DOI:** 10.1155/humu/9935937

**Published:** 2026-05-04

**Authors:** Zhi Li, Dedong Wang, Jinbin Chen, Di Wu, Shuyu Tang, Jinyi Huang, Yibin Deng, Xinhua Wang, Fuman Qiu, Lei Yang, Jiachun Lu

**Affiliations:** ^1^ The State Key Lab of Respiratory Disease, The First Affiliated Hospital, The Institute for Chemical Carcinogenesis, School of Public Health, Guangzhou Medical University, Guangzhou, Guangdong, China, gzhmc.edu.cn; ^2^ Guangzhou Center for Disease Control and Prevention, Guangzhou Health Supervision Institute, Guangzhou, Guangdong, China, chinacdc.cn; ^3^ Guangzhou Women and Children′s Medical Center, Guanzhou Medical University, Guangzhou Medical University, Guangzhou, Guangdong, China, gzhmc.edu.cn; ^4^ Centre for Medical Laboratory Science, the Affiliated Hospital of Youjiang Medical University for Nationalities, Baise, Guangxi, China, ymcn.gx.cn; ^5^ Institute of Basic Medicine, Institute of Public Health, Gansu University of Chinese Medicine, Lanzhou, Gansu, China, gszy.edu.cn

**Keywords:** glucose transporter 1, glycolytic, lung cancer, rs1385129, single nucleotide polymorphism

## Abstract

**Background:**

Energy metabolism reprogramming of cancer cells with the abnormal glycolytic capacity represents a novel direction of tumor therapy. Nevertheless, there is a lack of evidence linking genetic variations in glycolysis‐related genes to the risk and clinical progression of lung cancer (LC). This study is aimed at clarifying the genetic effect of glycolytic pathway‐related genes on the occurrence and development of LC.

**Methods:**

In this two‐stage case‐control study, we enrolled 300 LC patients and 600 healthy controls, as well as 1248 case‐control pairs from several hospitals in Guangzhou, to evaluate the association between the genetic variations of glycolysis‐related genes (*GLUT1* rs1385129G>A, *GLUT11* rs6003939A>C, *GLUT12* rs1484180G>A and *ENO2* rs11064467C>T) and the risk of LC. Follow‐up data and the TCGA database were used to evaluate the relationship between *GLUT1* rs1385129G>A and *GLUT1* expression with the clinical progression of LC.

**Results:**

Only *GLUT1* rs1385129G>A was found to be associated with increased risk of LC in this two‐stage case‐control study (*p* < 0.05). Further analysis of the expression levels of *GLUT1* in *GLUT1* rs1385129G>A genotypes showed that they were positively correlated with the number of A alleles (*p* < 0.01), and the GA genotype had a moderate effect on *GLUT1* expression, whereas the AA genotype had a strong effect (GA vs. GG: Cohen′s *d* = 0.768, 95*%* CI = 0.20–1.02; AA vs. GG: Cohen′s *d* = 1.890, 95*%* CI = 0.93–3.57). The results were further verified by eQTL analysis based on the GTEx database. The GA and AA genotypes were associated with worse prognosis in LC compared with the GG genotype, as determined by Cox regression (GA + AA vs. GG: HR = 1.37, 95*%* CI = 1.20–1.57). Furthermore, the survival curve of LC plotted using the GEPIA website showed that the group with high expression of *GLUT1* had an increased risk of poor prognosis compared with the low (Log‐rank *p* < 0.01; HR = 1.40). The same result was obtained from the Kaplan–Meier Plotter database (Log‐rank *p* < 0.01; HR = 1.34).

**Conclusion:**

Altogether, *GLUT1* rs1385129G>A may increase the risk of LC and contribute to a poor prognosis by upregulating *GLUT1* expression.

## 1. Introduction

Lung cancer (LC) is one of the most common cancers in the respiratory system, accounting for 11.6% of all cancer cases and representing the leading cause of cancer‐related deaths both in China and worldwide [1, 2], indicating that LC remains a severe global public health challenge. Studies have shown that tobacco is the most significant risk factor for LC, while air pollution, radiation, asbestos, arsenic, beryllium, and cadmium are also well‐established environmental risk factors [3, 4]. However, environmental risk factors are not the only contributors to the occurrence of LC. For instance, 15% of male and 53% of female LC patients are nonsmokers [5]. Furthermore, studies have demonstrated that LC results from a combination of genetic and environmental risk factors [6, 7].

Genome‐wide association studies (GWAS) have found a large number of susceptibility genes for LC in the past decade. For example, Niu et al. [8] reported that the *CHRNA3* rs3743073 polymorphism was associated with increased risk and worse prognosis of nonsmall cell lung cancer (NSCLC) in the Han Chinese population. Similarly, our previous studies [9, 10] identified genetic variant loci at 13q12.12 and 22q12.2 associated with the risk of LC, as well as loci at 6q21.1 and 7p15.3 linked to multiple cancer risks in the Han Chinese population. However, GWAS approaches are designed to identify the cancer‐related genes using extremely stringent significance thresholds, which may overlook subsignificant loci and fail to explain the overall heritability of cancers [11]. Therefore, it is critical to explore the association between functional variations in key candidate genes and LC susceptibility, with the aim of identifying genetic variant loci missed by GWAS.

Energy metabolism reprogramming is a typical characteristic of cancer cells [12]. Compared with normal cells, cancer cells reprogram the energy metabolism process, known as the “Warburg” effect, to provide raw materials and energy for continuous and unlimited cell proliferation, which is mainly manifested in the abnormal increase of glycolytic capacity, as well as the continuous production of energy through this inefficient pathway even under aerobic conditions [13]. Therefore, glycolytic pathway‐related genes play an important role in the occurrence and development of tumors. For example, glucose transporter 1 (*GLUT1*), also known as solute carrier family 2 facilitated glucose transporter member 1 (*SLC2A1*), is one of the key transport receptors in the process of cellular glycolysis. It has been shown that silencing the expression of *GLUT1* could inhibit the growth of tumor cells, whereas upregulation of *GLUT1* expression is associated with poor prognosis, metastasis, and tumor progression [14]. Therefore, we hypothesized that genetic variations in glycolysis‐related genes are linked to LC risk and progression. These variations may affect LC risk and prognosis by modulating target gene expression levels. However, few studies have investigated the association between genetic variations in glycolysis‐related genes and LC risk.

In this study, we conducted a two‐stage case‐control study and prognostic analysis to investigate the association between genetic variations in glycolysis‐related genes and LC risk and prognosis, aiming to clarify the genetic effects of glycolysis‐related genes on the occurrence and development of LC.

## 2. Materials and Methods

### 2.1. Study Subjects

We performed a two‐stage case‐control study in the Han Chinese based on several hospitals and communities in Guangzhou, China (Guangzhou Chest Hospital, The First Affiliated Hospital of Guangzhou Medical University, The Second Affiliated Hospital of Guangzhou Medical University, and The Affiliated Cancer Hospital and Institute of Guangzhou Medical University). In the first stage of the study, 300 LC patients and 600 healthy controls were enrolled from 2010 to 2019, and 1248 case‐control pairs were enrolled in the second stage from 2013 to 2019. All patients were confirmed by pathological diagnosis and had no previous history of malignant tumors.

All participants completed a questionnaire (including the demographic characteristics and risk factors such as smoking status and family history of cancer) to provide their demographic information and risk factors. Additionally, pathological and clinical staging information was obtained by reviewing their medical records. We collected 5 mL blood samples from each participant for genotyping and obtained 32 paracancerous tissues of LC patients among them for mRNA expression analysis after they signed the informed consent. Furthermore, we obtained prognostic information from LC patients through telephone follow‐ups conducted every 6 months from enrollment until December 2019.

The study was approved by the Ethics Committee of Guangzhou Medical University.

### 2.2. Candidate Genes

In this study, through extensive literature review and queries of the Reactome [15] and KEGG [16] databases, we identified genes related to key transporters and metabolic processes in glycolysis as candidate genes. These included glucose transporters (*GLUT*), hexokinases (*HK*), glucose‐6‐phosphate isomerase (*GPI*), phosphofructokinase (*PFK*), aldolase, glyceraldehyde‐3‐phosphate dehydrogenase (*GAPDH*), phosphoglycerate kinase (*PGK*), phosphoglycerate mutase (*PGM*), enolase (*ENO*), pyruvate kinase (*PK*), lactate dehydrogenase (*LDH*), and so on (shown in Table S1).

### 2.3. SNPs Selection

Using data from the Han Chinese in Beijing (CHB) population in Phase 3 of the 1000 Genomes Project (https://www.genome.gov/about-genomics), we extracted single nucleotide polymorphisms (SNPs) within the genomic regions and their 2 kb upstream and downstream flanking regions of the above candidate genes. We then retained loci with a minor allele frequency (MAF) greater than 0.05 that were located in exons, 5 ^′^‐UTR, and 3 ^′^‐UTR regions after preliminary screening. Subsequently, SNPs with potential functional effects were screened and analyzed using web servers such as RegulomeDB [17], 3DSNP [18], HaploReg [19], SNPinfo [20], and GTEx [21]. Finally, tag SNPs with potential biological functions that met Hardy–Weinberg equilibrium (HWE) and linkage disequilibrium (LD, *R* ^2^ < 0.8) criteria were retained as candidate loci.

A case‐control study with a small sample size was then conducted to preliminarily validate the candidate SNPs mentioned above for further screening. Using *α* = 0.01 as the significance level, Bonferroni correction was applied to adjust the threshold, resulting in an adjusted *α* ^′^ = 0.01/93 = 1.08 × 10^−4^. Finally, a total of 93 SNPs were included in the Phase I case‐control study (see Table S2), and 4 SNPs showed significant associations for further analysis (*GLUT1* rs1385129G>A, *GLUT11* rs6003939A>C, *GLUT12* rs1484180G>A, and *ENO2* rs11064467C > T, *p* < 1.08 × 10^−4^).

### 2.4. Genotyping and mRNA Expression Analysis

DNA was extracted from collected blood samples using the TIANamp Genomic DNA Kit (Tiangen Biotech, Beijing). Genotyping in the two‐stage case‐control study was performed using the MassARRAY system and TaqMan real‐time polymerase chain reaction (qPCR), respectively. Primers and probes were designed using Primer Express Software (Applied Biosystems, United States), and the details are listed in Table S3. In addition, a blank control was included on each plate, and 5% of the samples were randomly selected to verify the reliability of the qPCR results.

RNA extraction and reverse transcription into cDNA were performed using the BiooPureTM RNA Kit (Bioo Scientific, United States) and the PrimeScript RT reagent Kit (Takara, Japan) on paracancerous tissues from LC patients. The 7900HT system (Applied Biosystems, United States) was used to detect the expression of *GLUT1* by qPCR, and the primer information is listed in Table S4.

### 2.5. Expression Quantitative Trait Locus (eQTL) Analysis of SNPs and Prognostic Analysis of LC

To find out the association between genotype and gene expression, the eQTL analysis of SNPs was based on the cDNA from 32 paracancerous tissues of LC patients obtained above and the GTEx database [21] (https://www.gtexportal.org).

To explore the relationship between *GLUT1* expression level and prognosis of LC, prognostic analyses were conducted based on follow‐up data, the TCGA (https://portal.gdc.cancer.gov/), and the Kaplan–Meier Plotter databases (https://kmplot.com).

### 2.6. Statistical Analysis

The chi‐square test was used to compare the differences in demographic characteristics between the two groups, and the chi‐square goodness‐of‐fit test was used to evaluate HWE of allele frequency distribution of SNPs in controls.

The relationship between SNPs and the risk of LC was evaluated by logistic regression after adjusting for age, sex, smoking status, drinking status, family history of cancer, and LC history. Homogeneity across subgroups was evaluated using the Breslow‐Day test in the stratification analysis. Cohen′s d test was used to estimate the effect of SNPs on gene expression.

For prognostic association analysis, the log‐rank test was used to compare differences in demographic characteristics and survival time; the Cox regression model was used to calculate the hazard ratio (HR), and the Q test was used to analyze heterogeneity of subgroups. The Kaplan–Meier method was used to plot the survival curve.

Statistical analyses were performed using PLINK v1.90 (Harvard University, United States, http://pngu.mgh.harvard.edu/purcell/plink/), Stata 16 (StataCorp LLC, United States), IBM SPSS Statistics 25.0 software (IBM Corp., Chicago, Illinois, United States), and the Estimation Stats webserver (https://www.estimationstats.com/#/). The analysis of the Phase I case‐control study was two‐sided with statistical significance set at *p* < 0.01, and Bonferroni correction was used to adjust the statistics with an adjusted *α* ^′^ = *α*/93 = 1.08 × 10^−4^. All other tests were two‐sided and considered statistically significant at *p* < 0.05.

## 3. Results

### 3.1. Case‐Control Study

#### 3.1.1. Demographic Characteristics

Table S5 shows the demographic characteristics of the two‐stage case‐control study, and there were no significant differences between cases and controls in either stage of the study (*p* > 0.05), but significant differences were observed in family history of tumor and smoking status after combining the Phase I and Phase II case‐control studies (*p* < 0.05).

#### 3.1.2. Association Between SNPs and LC Risk

In the Phase I case‐control study, we conducted preliminary validation of the 93 candidate SNP loci identified through screening using a small sample size to further refine the selection of candidate loci. The results of the association analysis between SNPs and LC risk are listed in Table S6. The allele frequency distribution of the 93 SNPs was consistent with HWE in the controls (*P*_HWE_ > 0.01). Furthermore, we found that only the A allele of *GLUT1* rs1385129 (GG vs. GA vs. AA: OR = 1.65, 95% CI = 1.24–2.21, *p* = 7.75 × 10^−6^), the C allele of *GLUT11* rs6003939 (AA vs. AC vs. CC: OR = 2.31, 95*%* CI = 1.51–3.53, *p* = 3.49 × 10^−7^), the A allele of *GLUT12* rs1484180 (GG vs. GA vs. AA: OR = 2.27, 95*%* CI = 1.51–3.40, *p* = 1.85 × 10^−7^), and the T allele of *ENO2* rs11064467 (CC vs. CT vs. TT: OR = 1.85, 95*%* CI = 1.36–2.52, *p* = 2.57 × 10^−7^) were significantly associated with an increased risk of LC, and the risk increased with the number of risk alleles. Unfortunately, no significant association was found between the remaining 89 SNPs and the risk of LC (*p* > 1.08 × 10^−4^).

To further identify SNPs with significant associations, we analyzed the four SNPs mentioned above in Phase II and combined case‐control studies by expanding the sample size. As shown in Table 1, only *GLUT1* rs1385129G > A was found to be associated with increased risk of LC after adjusting for sex, age, smoking status, drinking status, family history of tumor and LC in phase II (AA vs. GG: OR = 1.53, 95*%* CI = 1.18–1.99, *p* = 0.001; GA + AA vs. GG: OR = 1.23, 95*%* CI = 1.05–1.44, *p* = 0.011), and no significant association was observed between the remaining SNPs and the risk of LC (*p* > 0.05). The same result was validated in the combined case‐control study (GA vs. GG: OR = 1.21, 95*%* CI = 1.05–1.40, *p* = 0.009; AA vs. GG: OR = 1.89, 95*%* CI = 1.50–2.39, *p* < 0.001; GA + AA vs. GG: OR = 1.32, 95*%* CI = 1.16–1.52, *p* < 0.001).

**Table 1:** Association of 4 SNPs with lung cancer risk in the Phase II and combined case‐control study.

	Phase II case‐control study				Combined case‐control study from Phase I and Phase II			
	Case *n* (%)	Control *n* (%)	OR (95% CI)^a^	*P*	Case *n* (%)	Control *n* (%)	OR (95% CI)^a^	*p*
	*n* = 124				*n* = 154			
Total	8	*n* = 1248			8	*n* = 1848		
***GLUT1 r*s1385129G>A**								
GG	560 (44.9)	621 (49.8)	1.00 (ref.)		690 (44.6)	952 (51.5)	1.00 (ref.)	
GA	524 (42.0)	508 (40.7)	1.16 (0.98–1.37)	0.090	654 (42.2)	748 (40.5)	1.21 (1.05–1.40)	0.009
AA	164 (13.1)	119 (9.5)	1.53 (1.18–1.99)	0.001	204 (13.2)	148 (8.0)	1.89 (1.50–2.39)	< 0.001
*P*_trend_				0.001				< 0.001
GA + AA versus GG	688 (55.1)	627 (50.2)	1.23 (1.05–1.44)	0.011	858 (55.4)	896 (48.5)	1.32 (1.16–1.52)	< 0.001
***GLUT11* rs6003939A>C**								
AA	1001 (80.2)	971 (77.8)	1.00 (ref.)		1212 (78.3)	1486 (80.4)	1.00 (ref.)	
AC	233 (18.7)	259 (20.8)	0.87 (0.71‐1.06)	0.159	319 (20.6)	340 (18.4)	1.15 (0.97–1.36)	0.120
CC	14 (1.1)	18 (1.4)	0.74 (0.37–1.51)	0.413	17 (1.1)	22 (1.2)	0.94 (0.50–1.79)	0.858
*P*_trend_				0.112				0.199
AC + CC versus AA	247 (19.8)	277 (22.2)	0.86 (0.71–1.04)	0.124	336 (21.7)	362 (19.6)	1.13 (0.96–1.34)	0.143
***GLUT12* rs1484180G>A**								
GG	967 (77.5)	936 (75)	1.00 (ref.)		1177 (76)	1444 (78.1)	1.00 (ref.)	
GA	252 (20.2)	288 (23.1)	0.84 (0.69–1.02)	0.076	334 (21.6)	376 (20.3)	1.09 (0.92–1.29)	0.310
AA	29 (2.3)	24 (1.9)	1.17 (0.68–2.03)	0.574	37 (2.4)	28 (1.5)	1.64 (0.99–2.69)	0.053
*P*_trend_				0.257				0.065
GA + AA versus GG	281 (22.5)	312 (25)	0.87 (0.72–1.04)	0.127	371 (24)	404 (21.9)	1.13 (0.96–1.32)	0.143
***ENO2* rs11064467C>T**								
CC	776 (62.2)	747 (59.9)	1.00 (ref.)		935 (60.4)	1155 (62.5)	1.00 (ref.)	
CT	400 (32.1)	423 (33.9)	0.91 (0.77–1.08)	0.290	512 (33.1)	598 (32.4)	1.06 (0.91–1.22)	0.467
TT	72 (5.8)	78 (6.3)	0.86 (0.62–1.21)	0.395	101 (6.5)	95 (5.1)	1.31 (0.98–1.76)	0.071
*P*_trend_				0.214				0.098
CT + TT versus CC	472 (37.8)	501 (40.1)	0.90 (0.77–1.06)	0.223	613 (39.6)	693 (37.5)	1.09 (0.95–1.25)	0.220

Abbreviations: CI, confidence interval; OR, odds ratio.

^a^ ORs were adjusted for age, sex, smoking status, drinking status, family history of tumor and lung cancer by the logistic regression model.

#### 3.1.3. Stratification and Interaction Analysis Between *GLUT1* rs1385129G>A and Risk of LC

Table 2 shows the results of stratification and interaction analysis between *GLUT1* rs1385129G>A and the risk of LC in the combined case‐control study. There were no differences among subgroups based on homogeneity test, and no multiplicative interaction was observed between the stratified factors and *GLUT1* rs1385129G>A on the risk of LC using logistic regression multiplication analysis (*P*_homo_ > 0.05 and *P*_inter_ > 0.05). Furthermore, except for the strata of drinking and family history of tumor or LC, GA, and AA remained risk factors in the strata when compared with the GG genotype (OR > 1, and 95% CI did not include 1).

**Table 2:** Stratification and interaction analysis between *GLUT1* rs1385129G>A and lung cancer risk in the combined case‐control study.

	Cases (*n* = 1548)			Controls (*n* = 1848)			*G* *A* + *A* *A*versus GG	*P*_*h* *o* *m* *o*_^b^	*P*_*i* *n* *t* *e* *r*_^c^
	GG, *n* (%)	GA, *n* (%)	AA, n (%)	GG, *n* (%)	GA, *n* (%)	AA, n (%)	Adjusted OR (95% CI)^a^		
Age									
≤ 60	382 (46.5)	333 (40.5)	107 (13.0)	507 (52.1)	396 (40.7)	71 (7.3)	1.25 (1.04–1.51)	0.438	0.266
> 60	445 (50.9)	352 (40.3)	77 (8.8)	308 (42.4)	321 (44.2)	97 (13.4)	1.41 (1.15–1.72)		

Sex									
Male	1155 (48.7)	976 (41.2)	240 (10.1)	667 (51.9)	516 (40.1)	103 (8.0)	1.33 (1.13–1.56)	0.957	0.848
Female	487 (47.5)	426 (41.6)	112 (10.9)	285 (50.7)	232 (41.3)	45 (8.0)	1.32 (1.03–1.69)		

Smoking status									
NO	297 (43.4)	298 (43.6)	89 (13)	465 (51.7)	362 (40.3)	72 (8.0)	1.40 (1.15–1.72)	0.464	0.693
YES	393 (45.5)	356 (41.2)	115 (13.3)	487 (51.3)	386 (40.7)	76 (8.0)	1.25 (1.04–1.51)		

Drinking status									
NO	544 (44.5)	512 (41.9)	166 (13.6)	765 (52.2)	586 (40)	114 (7.8)	1.36 (1.17–1.59)	0.353	0.647
YES	146 (44.8)	142 (43.6)	38 (11.7)	187 (48.8)	162 (42.3)	34 (8.9)	1.19 (0.88–1.61)		

Family history of tumor									
NO	615 (44.4)	584 (42.2)	185 (13.4)	876 (51.8)	689 (40.7)	127 (7.5)	1.35 (1.17–1.55)	0.844	0.275
YES	75 (45.7)	70 (42.7)	19 (11.6)	76 (48.7)	59 (37.8)	21 (13.5)	1.13 (0.72–1.75)		

Family history of lung cancer									
NO	674 (44.7)	636 (42.1)	199 (13.2)	931 (51.7)	729 (40.5)	142 (7.9)	1.33 (1.16–1.52)	0.822	0.747
YES	16 (41.0)	18 (46.2)	5 (12.8)	21 (45.7)	19 (41.3)	6 (13.0)	1.22 (0.49–2.99)		

Abbreviations: CI, confidence interval; OR, odds ratio.

^a^ORs were adjusted for age, sex, smoking status, drinking status, family history of tumor and lung cancer by the logistic regression model.

^b^ *p* value of the Breslow‐day homogeneity test for the ORs between strata.

^c^ *p* value of test for the multiplicative interaction between *GLUT1* rs1385129 genotypes and the factors.

#### 3.1.4. eQTL Analysis of GLUT1 rs1385129G>A

**Figure 1:**
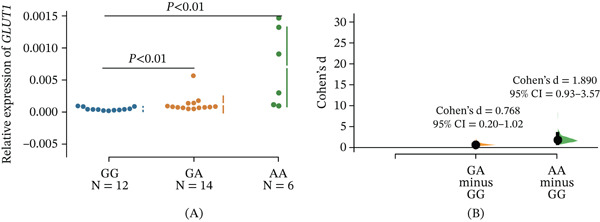
(A) The association between GLUT1 rs1385129G>A and GLUT1 expression level in 32 paracancerous tissues from lung cancer patients; (B) Cohen′s d effect size of GLUT1 rs1385129G>A.

To explore the relationship between *GLUT1* rs1385129G>A and the expression level of *GLUT1*, we detected *GLUT1* expression using qPCR on 32 paracancerous tissues from LC patients. As shown in Figure 1A, the expression level of *GLUT1* in GA and AA genotypes was significantly higher than in the GG genotype and positively correlated with the number of A alleles (*p* < 0.01). Then, the Cohen′s d effect size analysis showed that the GA genotype had a moderate effect on *GLUT1* expression and the AA genotype had a strong effect compared with the GG genotype (GA vs. GG: Cohen′s *d* = 0.768, 95*%* CI = 0.20–1.02; AA vs. GG: Cohen′s *d* = 1.890, 95*%* CI = 0.93–3.57; Figure 1B).

**Figure 2:**
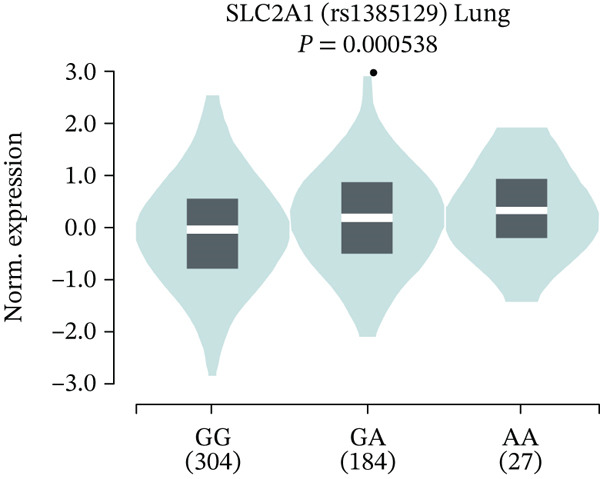
eQTL analysis of *GLUT1* rs1385129G>A based on the GTEx database.

Furthermore, we performed an eQTL analysis of *GLUT1* rs1385129G>A in lung tissues based on data from 515 healthy individuals in the GTEx database to verify its effect on *GLUT1* expression. Similarly, the results were consistent with those from the 32 paracancerous tissues of LC patients [(median) GG vs. GA vs. AA: −0.110 vs. 0.154 vs. 0.301, *p* < 0.001, Figure 2].

#### 3.1.5. Association Between *GLUT1* rs1385129G>A and Clinical Progression of LC

Table 3 shows the results of the association between *GLUT1* rs1385129G>A and clinical stages of LC, and clinical progression of T, N, M stages in the combined case‐control study. Compared with the GG genotype, the GA and AA genotypes of rs1385129 showed significant differences in clinical stages, and T, N, M stages of LC, which were associated with worse clinical progression (clinical stage: III + IV vs. I + II: OR = 1.54, 95*%* CI = 1.23–1.93, *p* < 0.001; T stage: T3 + T4 vs. T1 + T2: *OR* = 1.47, 95*%* CI = 1.20–1.80, *p* < 0.001; N stage: N1 + N2 + N3 vs. N0: OR = 1.32, 95*%* CI = 1.05–1.66, *p* = 0.019; M stage: M1 vs.M0:OR = 1.32, 95*%* CI = 1.08–1.62, *p* = 0.007).

**Table 3:** Association of *GLUT1* rs1385129G > A with clinical progression of lung cancer.

	rs1385129G > A			GA + AA versus GG	*p*
	GG, *n* (%)	GA, *n* (%)	AA, *n* (%)	Adjusted OR (95% CI)^a^	
Clinical stage					
I + II	218 (31.6)	148 (22.6)	50 (24.5)	1.00 (ref.)	< 0.001
III + IV	472 (68.4)	506 (77.4)	154 (75.5)	1.54 (1.23–1.93)	
T stage					
T1 + T2	370 (53.6)	281 (43.0)	97 (47.5)	1.00 (ref.)	< 0.001
T3 + T4	320 (46.4)	373 (57.0)	107 (52.5)	1.47 (1.20–1.80)	
N stage					
N0	197 (28.6)	150 (22.9)	50 (24.5)	1.00 (ref.)	0.019
N1 + N2 + N3	493 (71.4)	504 (77.1)	154 (75.5)	1.32 (1.05–1.66)	
M stage					
M0	418 (60.6)	349 (53.4)	113 (55.4)	1.00 (ref.)	0.007
M1	272 (39.4)	305 (46.6)	91 (44.6)	1.32 (1.08–1.62)	

Abbreviations: CI, confidence interval; OR, odds ratio.

^a^ORs were adjusted for age, sex, smoking status, drinking status, family history of tumor and lung cancer by the logistic regression model.

### 3.2. Association Between *GLUT1* rs1385129G>A and the Prognosis of LC

#### 3.2.1. *GLUT1* rs1385129G>A Is Associated With Poor Prognosis in LC

We followed up a total of 1190 patients out of 1548 cases by December 2019, and 956 (80.3%) of them reached the expected endpoint (death). As shown in Table 4, there were significant differences in median survival time (MST) among follow‐up subjects with GG, GA, and AA genotypes (Log‐rank *p* < 0.001). Individuals with the GG genotype had a significantly longer MST (21 months) than those with GA or AA genotypes (14 months each). Moreover, patients with GA and AA genotypes had a worse prognosis of LC compared with those with the GG (GA vs. GG: HR = 1.31, 95*%* CI = 1.14–1.51; AA vs. GG: HR = 1.60, 95*%* CI = 1.14–1.51; GA + AA vs. GG: HR = 1.37, 95*%* CI = 1.20–1.57).

**Table 4:** Association between *GLUT1* rs1385129G > A and the prognosis of lung cancer among 1190 follow‐up subjects.

SNP	Case *n* (%)	Expected outcome (death) *n* (%)	MST (months)	Log‐rank *P*	HR (95% CI)^a^
rs1385129 G>A	1190	956 (80.3)			
GG	526 (44.2)	380 (39.7)	21	< 0.001	1.00 (ref.)
GA	508 (42.7)	441 (46.1)	14		1.31 (1.14–1.51)
AA	156 (13.1)	135 (14.1)	14		1.60 (1.31–1.95)
*P*_trend_					<0.001
GA + AA	664 (55.8)	576 (60.3)	14	< 0.001	1.37 (1.20–1.57)

Abbreviations: HR, hazard ratio; MST, median survival time.

^a^HRs were adjusted for age, sex, smoking status, drinking status, family history of tumor and lung cancer, surgery, chemotherapy, radiotherapy, clinical stages and histological classification by the cox regression model.

#### 3.2.2. Stratification and Interaction Analysis Between *GLUT1* rs1385129G > A and Prognosis of LC

Table 5 shows the results of the stratification and interaction analysis between *GLUT1* rs1385129G>A and the LC prognosis among 1190 follow‐up subjects above. There was heterogeneity in the prognosis of LC among different subgroups of clinical stage (*P*_homo_ = 0.044), and there was a multiplicative interaction between clinical stage and rs1385129G>A on the prognosis of LC (*P*_inter_ = 0.029). Furthermore, compared with the GG genotype, the GA and AA genotypes remained associated with increased risk in the strata of factors except drinking, family history of tumor or LC, without chemotherapy, I + II of clinical stage, large cell carcinoma, and other histological types (HR > 1 and 95% CI did not include 1).

**Table 5:** Stratification and interaction analysis between *GLUT1* rs1385129G > A and the prognosis of lung cancer among 1190 follow‐up subjects.

	GG (*n*)		GA (*n*)		GA (*n*)		*G* *A* + *A* *A* versus GG	*P*_homo_^b^	*P*_inter_^c^
	Case	Expected outcome (death)	Case	Expected outcome (death)	Case	Expected outcome (death)	HR (95% CI)^a^		
Age									
≤ 60	289	204	257	220	87	76	1.30 (1.08–1.57)	0.424	0.344
> 60	237	176	251	221	69	59	1.45 (1.20–1.76)		

Sex									
Male	381	272	363	306	105	95	1.33 (1.14–1.56)	0.268	0.545
Female	145	108	145	135	51	40	1.57 (1.23–2.02)		

Smoking status									
NO	219	163	233	212	63	47	1.34 (1.09–1.64)	0.673	0.661
YES	307	217	275	229	93	88	1.42 (1.19–1.69)		

Drinking status									
NO	413	300	401	354	126	106	1.39 (1.20–1.61)	0.759	0.735
YES	113	80	107	87	30	29	1.32 (0.98–1.77)		

Family history of tumor									
NO	467	338	455	397	142	122	1.39 (1.21–1.60)	0.788	0.735
YES	59	42	53	44	14	13	1.48 (0.96–2.29)		

Family history of lung cancer									
NO	512	368	492	429	153	132	1.40 (1.22–1.60)	0.177	0.170
YES	14	12	16	12	3	3	0.68 (0.24–1.92)		

Surgery									
NO	234	174	252	226	83	70	1.34 (1.11–1.63)	0.871	0.95
YES	292	206	256	215	73	65	1.37 (1.14–1.65)		

Chemotherapy									
NO	176	133	180	153	49	42	1.21 (0.96–1.52)	0.163	0.159
YES	350	247	328	288	107	93	1.48 (1.25–1.74)		

Radiotherapy									
NO	218	156	209	187	71	59	1.46 (1.19–1.80)	0.433	0.389
YES	308	224	299	254	85	76	1.31 (1.10–1.56)		

Clinical Stage									
I + II	162	125	106	57	35	20	1.05 (0.78–1.41)	0.044	0.029
III + IV	364	255	402	384	121	115	1.48 (1.27–1.73)		

Histological classification									
Adenocarcinoma	284	208	248	221	83	73	1.37 (1.14–1.64)	0.168	0.798
Squamous cell carcinoma	164	116	169	140	52	45	1.44 (1.13–1.83)		
Large cell carcinoma	14	11	12	9	6	5	0.41 (0.15–1.11)		
Small cell carcinoma	48	36	66	60	12	9	1.67 (1.06–2.64)		
Others^d^	16	9	13	11	3	3	1.48 (0.36–6.17)		

Abbreviation: HR, hazard ratio.

^a^HRs were adjusted for age, sex, smoking status, drinking status, family history of tumor and lung cancer, surgery, chemotherapy, radiotherapy, clinical stages and histological classification by the Cox regression model.

^b^ *p* value of the Q test for the HRs between strata.

^c^ *p* value of test for the multiplicative interaction between *GLUT1* rs1385129 genotypes and the factors.

^d^Mixed or undifferentiated carcinoma.

#### 3.2.3. Association Between *GLUT1* Expression and Prognosis of LC Based on TCGA and Kaplan–Meier Plotter

**Figure 3:**
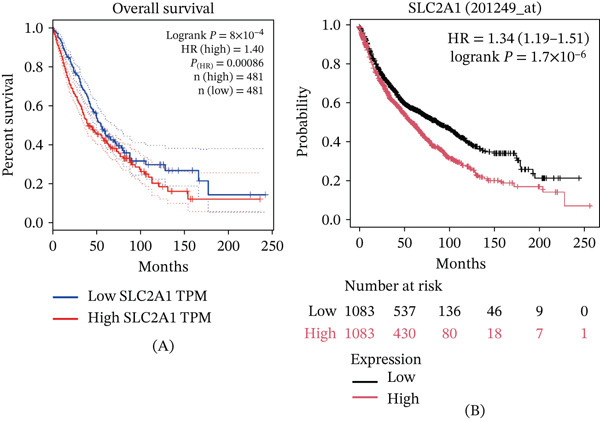
(A) The survival curve of LUAD and LUSC samples from the TCGA database; (B) survival analysis of 2166 lung cancer patients with prognostic information in the Kaplan–Meier Plotter database. [GLUT1 is also known as the solute carrier family 2 facilitated glucose transporter member 1 (SLC2A1)].

We plotted the survival curve of lung adenocarcinoma (LUAD) and squamous cell carcinoma (LUSC) samples from TCGA using the GEPIA website (http://gepia.cancer-pku.cn/index.html) (shown in Figure 3A). It showed that the group with high expression of *GLUT1* had an increased risk of poor prognosis compared with the low (Log‐rank *p* < 0.01; HR = 1.40). In addition, the same result was obtained through survival analysis of 2166 LC patients with prognostic information in the Kaplan–Meier Plotter database (https://kmplot.com/) (Log‐rank *p* < 0.01; HR = 1.34, 95% CI = 1.19 − 1.51; Figure 3B).

## 4. Discussion

LC is one of the malignant tumors with the highest morbidity and mortality worldwide, as well as the main cause of tumor‐related death [22, 23]. With environmental pollution, lifestyle changes, occupational exposure, and increasing tobacco consumption, LC has shown a substantial increase in incidence, mortality and disease burden over the last decades in China [24]. What is more, it has become a global public health problem due to the late diagnosis and related poor treatment measures with an overall 5‐year survival rate of only 10%–20%. Current studies have shown that energy metabolism reprogramming is one of the characteristics of tumors, which is mainly manifested by the abnormal increase in energy production through glycolysis under aerobic conditions, also known as the “Warburg” effect [25, 26]. In this study, we found that *GLUT1* rs1385129G>A, a genetic variation of glycolytic pathway‐related genes, was associated with the risk and prognosis of LC, where the A allele conferred an increased risk of poor progression.

*GLUT1* is a protein that promotes the transmembrane transport of glucose, which is a key protein in the cellular energy metabolism pathway. It was found to be overexpressed in cancer and reported to promote metastasis in cancer cells [27]. Studies have demonstrated that *GLUT1* acts as a key regulator in different types of tumors, such as prostate cancer [28], glioblastoma [29], breast cancer [30], osteosarcoma [31], esophageal squamous cell carcinoma [32], and melanoma [33]. Furthermore, Liu et al. [34] reported that upregulation of *GLUT1* might be a key mechanism of glucose uptake to support the energy requirements of cancer cells. For example, mRNA and protein expression levels of *GLUT1* were upregulated in LC tissues compared with normal lung epithelial cells. Similarly, in this study, we found that high expression of *GLUT1* was associated with an increased risk of poor prognosis in LC, which is consistent with previous findings. The above results suggest that *GLUT1* acts as a key regulator of energy metabolism and plays an important role in the occurrence and development of not only LC but also other tumors, which could be a biomarker for predicting clinical progression and prognosis in cancer [35].

Rs1385129G>A is a synonymous variant located in exon 2 of *GLUT1* and acts as an enhancer. An enhancer is a key regulatory element of DNA, which plays an important role in gene transcription regulation [36]. The sequence mutation of it could disrupt the enhancer–promoter loop and affect their interactions, which may underlie disease susceptibility [37]. Current studies have reported that rs1385129G>A is associated with diabetic nephropathy [38, 39], and could predict the progression of HIV infection [40]. Although a large number of studies have found that *GLUT1* expression and genetic variation are associated with clinical progression and prognosis of various tumors, *GLUT1* rs1385129G>A has not been reported. In this study, we found that *GLUT1* rs1385129G>A was associated with the risk of LC and the risk increased with the number of A alleles. Furthermore, the eQTL analysis of this SNP in 32 paracancerous tissues from LC patients and 515 lung tissues from the GTEx database suggested that the expression level of *GLUT1* in the AA genotype was significantly higher than that in the GG and GA, indicating that the rs1385129G>A variant increases the risk of LC by upregulating the expression of *GLUT1*. Additionally, the prognostic analysis showed that the variant contributes to poor LC progression by promoting the high expression of *GLUT1*. These results suggest that*GLUT1* rs1385129G>A is a potential biomarker for susceptibility and prognosis of LC.

Despite our careful design, there are some limitations to this study. First, its hospital‐based case‐control design, which relies on hospital data, inevitably introduces potential biases, such as selection bias and recall bias. Second, further biological functional assays and animal experiments are required to elucidate the biological mechanism by which *GLUT1* rs1385129G>A affects the occurrence and development of LC.

In conclusion, we systematically investigated the association between genetic variants in glycolysis‐related genes and the risk of LC in this two‐stage case‐control study and demonstrated that *GLUT1* rs1385129G>A is associated with an increased risk and poor prognosis of LC. Specifically, as the number of rs1385129 A alleles increased, *GLUT1* expression levels were correspondingly upregulated, and the risk of LC increased. These findings suggest that *GLUT1* rs1385129G>A may increase LC risk and lead to poor prognosis of LC by upregulating the expression of *GLUT1*, which can serve as a potential molecular biomarker for lung cancer risk prediction and prognostic evaluation.

**Nomenclature**
GWAS: genome‐wide association study
NSCLC: non‐small cell lung cancer
GLUT1: glucose transporter 1
SNPs: single nucleotide polymorphisms
HWE: Hardy–Weinberg equilibrium
qPCR: real‐time polymerase chain reaction
eQTL: expression quantitative trait locus
HR: hazard ratio
OR: odds ratio
CI: confidence interval
MST: median survival time
LUAD: lung adenocarcinoma
LUSC: lung squamous cell carcinoma

## Author Contributions

Dedong Wang conceived and designed the study with Jiachun Lu. Zhi Li and Di Wu wrote and modified the manuscript. Dedong Wang and Di Wu performed the experiments. Zhi Li and Jinbin Chen collected and analyzed the data. Shuyu Tang, Fuman Qiu, Lei Yang, Yibin Deng, and Xinhua Wang provided help and advice on manuscript review and editing. Jinyi Huang participated in the manuscript modifications and statistical review. Zhi Li, Dedong Wang, Jinbin Chen, and Di Wu contributed equally to this work and should be regarded as co‐first authors.

## Funding

This study was supported by National Natural Science Foundation of China (10.13039/501100001809; 82173609, 82373678, 81803325, 81803325); Natural Science Foundation of Guangdong Province (10.13039/501100003453, 2021A1515011175).

## Disclosure

All authors read and approved the final manuscript.

## Ethics Statement

The study was approved by the Ethics Committee of Guangzhou Medical University. Informed consent was obtained from all participants.

## Consent

The authors have nothing to report.

## Conflicts of Interest

The authors declare no conflicts of interest.

## Supporting information


**Supporting Information** Additional supporting information can be found online in the Supporting Information section. Table S1 Candidate genes of the glycolysis pathway. Table S2: Details of 93 SNPs in the Phase I case‐control study. Table S3: Sequences of TaqMan genotyping primers and probes. Table S4: The qRT‐PCR primers of GLUT1. Table S5: Demographic characteristics of the case‐control study. Table S6: Association of SNPs with lung cancer risk in the Phase I case‐control study.

## Data Availability

The dataset analyses during the current study are available in the GTEx database (https://www.gtexportal.org/home/testyourown) with the parameter of “rs1385129, ENSG00000117394.20, Lung”; the GEPIA website (http://gepia.cancer-pku.cn/detail.php?gene=SLC2A1%26clicktag=survival) with the datasets of “LUAD and LUSC” of SLC2A1 gene; and the Kaplan–Meier Plotter database (https://kmplot.com/analysis/index.php?p=service%26cancer=lung) with the gene symbol “201249_at (SLC2A1).” The data that support the findings of this study are available from the corresponding author upon reasonable request.
