# Supplementary material for: GLUT1 rs1385129G>A Raised the Risk and Poor Prognosis of Lung Cancer: A Case‐Control Study
Source: Hum Mutat. 2026 May 4;2026:9935937. doi: 10.1155/humu/9935937 (PMC13139712; doi:10.1155/humu/9935937)
Supplement: Supplementary file 1 — Supporting Information Additional supporting information can be found online in the Supporting Information section. Table S1 Candidate genes of the glycolysis pathway. Table S2: Details of 93 SNPs in the Phase I case‐control study. Table S3: Sequences of TaqMan genotyping primers and probes. Table S4: The qRT‐PCR primers of GLUT1. Table S5: Demographic characteristics of the case‐control study. Table S6: Association of SNPs with lung cancer risk in the Phase I case‐control study. [file HUMU-2026-9935937-s001.docx]

| **Supplementary Table 1.** **Candidate genes of the glycolysis pathway** | |
| --- | --- |
| **Transporter and metabolic enzyme family** | **Candidate genes** |
| Glucose Transporter | *GLUT1、GLUT2、GLUT3、GLUT4、GLUT5、GLUT6、GLUT7、GLUT8、GLUT9、GLUT10、GLUT11、GLUT12、GLUT13、**GLUT14* |
| hexokinase | *HK1、HK2、HK3* |
| 6-phosphate glucose isomerase | *GPI* |
| 6-phosphofructokinase | *PFKP、PFKL、PFKM* |
| Fructose-Bisphosphate Aldolase | *ALDOA、ALDOB、ALDOC* |
| glyceraldehyde-3-phosphate dehydrogenase | *GAPDH* |
| Phosphoglycerate kinase | *PGK2* |
| phosphoglycerate mutase | *PGAM1、PGAM2、PGAM5* |
| enolase | *ENO1、ENO2、ENO3、ENO4* |
| pyruvate kinase | *PKM、PKLR* |
| lactate dehydrogenase | *LDHA、LDHB、LDHC、LDHD* |

| **Supplementary Table 2. Details of 93 SNPs in the phase Ⅰ case-control study** | | |
| --- | --- | --- |
| **Genes and SNPs** | **Reference/ Effector allele** | **Annotation** |
| *GLUT1* |  |  |
| rs1385129 | G/A | synonymous variant |
| rs2229682 | C/T | synonymous variant |
| rs11537640 | T/G | 5’-UTR variant |
| *GLUT2* |  |  |
| rs5398 | C/T | synonymous variant |
| *GLUT3* |  |  |
| rs73059359 | T/A | 5’-UTR variant |
| rs7966327 | A/G | 5’-UTR variant |
| *GLUT4* |  |  |
| rs5417 | C/A | 5’-UTR variant |
| rs5415 | C/T | 5’-UTR variant |
| rs5412 | G/A | 5’-UTR variant |
| *GLUT5* |  |  |
| rs1063137 | G/A | 3’-UTR variant |
| rs6658278 | G/A | 5’-UTR variant |
| rs770041 | G/A | 5’-UTR variant |
| rs12117043 | G/A | 5’-UTR variant |
| rs3820034 | C/T | 5’-UTR variant |
| rs34488100 | T/G | 3’-UTR variant |
| rs1060998 | C/T | 3’-UTR variant |
| *GLUT6* |  |  |
| rs3124757 | A/G | 5’-UTR variant |
| *GLUT7* |  |  |
| rs12032857 | C/G | 5’-UTR variant |
| rs10864379 | G/A | synonymous variant |
| rs72632913 | G/A | 5’-UTR variant |
| rs34545462 | C/T | missense variant |
| *GLUT8* |  |  |
| rs3808835 | A/C | 5’-UTR variant |
| rs5900747 | C/A | 3’-UTR variant |
| *GLUT9* |  |  |
| rs10939650 | T/C | synonymous variant |
| rs6820230 | C/T | missense variant |
| rs3733589 | C/T | synonymous variant |
| rs4621429 | A/G | 3’-UTR variant |
| rs3733591 | A/G | missense variant |
| *GLUT10* |  |  |
| rs707507 | C/T | 3’-UTR variant |
| rs6094435 | C/T | 5’-UTR variant |

| **Supplementary Table 2. Details of 93 SNPs in the phase Ⅰ case-control study (continue)** | | |
| --- | --- | --- |
| **Genes and SNPs** | **Reference/ Effector allele** | **Annotation** |
| rs2235491 | G/A | missense variant |
| rs6017995 | A/G | 5’-UTR variant |
| *GLUT11* |  |  |
| rs6003939 | A/C | 3’-UTR variant |
| rs1129067 | C/T | 3’-UTR variant |
| *GLUT12* |  |  |
| rs1484180 | G/A | 5’-UTR variant |
| rs2200285 | G/A | 3’-UTR variant |
| rs1385066 | T/C | 3’-UTR variant |
| *GLUT14* |  |  |
| rs7309043 | G/A | 5’-UTR variant |
| rs1894824 | C/T | 3’-UTR variant |
| *HK1* |  |  |
| rs76042857 | G/A | 5’-UTR variant |
| rs748235 | A/G | synonymous variant |
| rs1108272 | C/T | splice region variant, |
| rs1133189 | C/G | synonymous variant |
| rs10998710 | C/A | 5’-UTR variant |
| *HK2* |  |  |
| rs2229627 | C/G | synonymous variant |
| rs10194657 | A/G | synonymous variant |
| rs2229626 | C/T | synonymous variant |
| rs656489 | G/A | 5’-UTR variant |
| rs2229622 | T/C | synonymous variant |
| *HK3* |  |  |
| rs2278492 | C/T | synonymous variant |
| rs59411283 | C/T | 5’-UTR variant |
| *PFKP* |  |  |
| rs1052333 | C/T | synonymous variant |
| rs541 | C/T | 3’-UTR variant |
| rs1132173 | C/T | synonymous variant |
| rs10795005 | T/C | 5’-UTR variant |
| rs4881086 | C/T | synonymous variant |
| rs10903966 | T/C | 5’-UTR variant |
| rs4881106 | G/T | 3’-UTR variant |
| rs10903967 | G/A | splice region variant, |
| rs2306302 | G/A | synonymous variant |
| rs4256893 | G/A | 5’-UTR variant |
| rs11251720 | T/C | 5’-UTR variant |
| rs3816704 | G/A | 3’-UTR variant |
| rs35863365 | C/T | 5’-UTR variant |

| **Supplementary Table 2. Details of 93 SNPs in the phase Ⅰ case-control study (continue)** | | |
| --- | --- | --- |
| **Genes and SNPs** | **Reference/ Effector allele** | **Annotation** |
| rs79334726 | G/A | 3’-UTR variant |
| rs34543032 | T/C | 5’-UTR variant |
| rs4269839 | T/G | 5’-UTR variant |
| *PFKL* |  |  |
| rs2847227 | T/C | 5’-UTR variant |
| *PFKM* |  |  |
| rs11168418 | C/A | 5’-UTR variant |
| rs1049392 | C/T | synonymous variant |
| *ALDOB* |  |  |
| rs4577 | C/T | 3’-UTR variant |
| *ALDOC* |  |  |
| rs652677 | G/T | 5’-UTR variant |
| *GAPDH* |  |  |
| rs6489721 | T/C | 5’-UTR variant |
| rs7971637 | C/T | 5’-UTR variant |
| *PGAM5* |  |  |
| rs11615090 | T/C | 5’-UTR variant |
| rs12423651 | C/T | missense variant |
| rs4883606 | C/T | 3’-UTR variant |
| rs117103262 | T/C | 3’-UTR variant |
| *ENO1* |  |  |
| rs2274971 | A/G | 5’-UTR variant |
| rs6660137 | A/G | 5’-UTR variant |
| rs17032801 | A/G | 5’-UTR variant |
| *ENO2* |  |  |
| rs11064467 | C/T | 3’-UTR variant |
| rs2238116 | A/G | 5’-UTR variant |
| rs710416 | A/C | 5’-UTR variant |
| *ENO3* |  |  |
| rs112089924 | C/T | 5’-UTR variant |
| rs238238 | A/G | missense variant |
| *ENO4* |  |  |
| rs9421246 | C/T | 5’-UTR variant |
| rs11197835 | A/C | synonymous variant |
| rs2257791 | A/G | 3’-UTR variant |
| *PKM* |  |  |
| rs12372964 | T/C | 5’-UTR variant |
| *LDHA* |  |  |
| rs2249631 | G/A | 5’-UTR variant |
| rs7129689 | T/C | 5’-UTR variant |
| *LDHC* |  |  |
| rs72868553 | C/A | 5’-UTR variant |

| **Supplementary Table 3. Sequences of TaqMan genotyping primers and probes** | | |
| --- | --- | --- |
| **SNPs** | **primer and probe** | **Sequences (5’-3’)** |
| rs1385129G>A | Forward Prime | ACTGCAGGGAGCCAAGCA |
|  | Reverse Prime | TGCTCCCAGACACGCCTATAA |
|  | probe1 | CCCACGGCCAGCA |
|  | probe2 | TCCTCCCACAGCC |
| rs6003939A>C | Forward Prime | TCCAGGTGCTTAGCAATCAATG |
|  | Reverse Prime | CTGCCACAACCAGCCTGTAAT |
|  | probe1 | ACAGAAAATCAGTAACCACAT |
|  | probe2 | CAGAAAATCAGTAACAACAT |
| rs1484180G>A | Forward Prime | TTGGCTACACAAAACAGAGTGAGAA |
|  | Reverse Prime | CATGGGAGTGTTAGAGTGTTTGCT |
|  | probe1 | CTGTCTAGTCAAGCACT |
|  | probe2 | TCTGTCTAGTCAAGTACT |
| rs11064467C>T | Forward Prime | GTTGGTGTGCTGAGGTGTTAGAGA |
|  | Reverse Prime | TATGCACAGTTCACGGCTCATAT |
|  | probe1 | CTCTTGTCCCACGTGT |
|  | probe2 | CTCTTGTTCCACGTGTC |

| **Supplementary Table 4. The qRT-PCR primers of *GLUT1*** | |
| --- | --- |
|  | **Sequences (5’-3’)** |
| Forward Prime | TCTGGCATCAACGCTGTCTTC |
| Reverse Prime | CGATACCGGAGCCAATGGT |

| **Supplementary Table 5. Demographic characteristics of the case-control study** | | | | | | | | | | | |
| --- | --- | --- | --- | --- | --- | --- | --- | --- | --- | --- | --- |
| **Variables** | **the phase I case-control study** | | |  | **the phase II case-control study** | | |  | **the combined case-control study** | | |
|  | **Case (n=300)**  **n (%)** | **Control (n=600) n (%)** | ***P ^a^*** |  | **Case (n=1248) n (%)** | **Control (n=1248) n (%)** | ***P ^a^*** |  | **Case (n=1548) n (%)** | **Control (n=1848) n (%)** | ***P* ^a^** |
| Age |  |  |  |  |  |  |  |  |  |  |  |
| ≤60 | 175 (58.3) | 361 (60.2) | 0.597 |  | 647 (51.8) | 613 (49.1) | 0.173 |  | 822 (53.1) | 974 (52.7) | 0.818 |
| >60 | 125 (41.7) | 239 (39.8) |  |  | 601 (48.2) | 635 (50.9) |  |  | 726 (46.9) | 874 (47.3) |  |
| Sex |  |  |  |  |  |  |  |  |  |  |  |
| Male | 210 (70.0) | 403 (67.2) | 0.390 |  | 875 (70.1) | 883 (70.8) | 0.726 |  | 1085 (70.1) | 1286 (69.6) | 0.751 |
| female | 90 (30.0) | 197 (32.8) |  |  | 373 (29.9) | 365 (29.2) |  |  | 463 (29.9) | 562 (30.4) |  |
| Family history of tumor |  |  |  |  |  |  |  |  |  |  |  |
| YES | 29 (9.7) | 45 (7.5) | 0.265 |  | 135 (10.8) | 111 (8.9) | 0.107 |  | 164 (10.6) | 156 (8.4) | 0.032 |
| NO | 271 (90.3) | 555 (92.5) |  |  | 1113 (89.2) | 1137 (91.1) |  |  | 1384 (89.4) | 1692 (91.6) |  |
| Family history of lung cancer |  |  |  |  |  |  |  |  |  |  |  |
| YES | 7 (2.3) | 11 (1.8) | 0.614 |  | 32 (2.6) | 35 (2.8) | 0.710 |  | 39 (2.5) | 46 (2.5) | 0.955 |
| NO | 293 (97.7) | 589 (98.2) |  |  | 1216 (97.4) | 1213 (97.2) |  |  | 1509 (97.5) | 1802 (97.5) |  |
| Smoking status |  |  |  |  |  |  |  |  |  |  |  |
| YES | 163 (54.3) | 293 (48.8) | 0.120 |  | 701 (56.2) | 656 (52.6) | 0.071 |  | 864 (55.8) | 949 (51.4) | 0.009 |
| NO | 137 (45.7) | 307 (51.2) |  |  | 547 (43.8) | 592 (47.4) |  |  | 684 (44.2) | 899 (48.6) |  |
| Drinking status |  |  |  |  |  |  |  |  |  |  |  |
| YES | 70 (23.3) | 114 (19.0) | 0.129 |  | 256 (20.5) | 269 (21.6) | 0.523 |  | 326 (21.1) | 383 (20.7) | 0.811 |
| NO | 230 (76.7) | 486 (81.0) |  |  | 992 (79.5) | 979 (78.4) |  |  | 1222 (78.9) | 1465 (79.3) |  |
| ^a^ Chi-square test | | | | | | | | | | | |

| **Supplementary Table 6. Association of SNPs with lung cancer risk in the phase I case-control study** | | | | | | |
| --- | --- | --- | --- | --- | --- | --- |
| **SNPs** | **Genotypes (WT/Het/Hom)^a^** | | **MAF** | ***P*_HWE_** | **adjust**  **OR (95% CI)^b^** | ***P* ^c^** |
|  | **Case (n=300)** | **Control (n=600)** |  |  |  |  |
| *GLUT1* |  |  |  |  |  |  |
| rs1385129G>A | 130/130/40 | 331/240/29 | 0.25 | 0.100 | 1.65 (1.24-2.21) | 7.75×10^-6^ |
| rs2229682C>T | 263/34/3 | 510/84/6 | 0.08 | 0.258 | 0.82 (0.5-1.34) | 0.288 |
| rs11537640T>G | 170/108/22 | 398/187/15 | 0.18 | 0.269 | 1.53 (1.11-2.10) | 0.001 |
| *GLUT2* |  |  |  |  |  |  |
| rs5398C>T | 178/101/21 | 356/216/28 | 0.23 | 0.562 | 1.06 (0.78-1.44) | 0.602 |
| *GLUT3* |  |  |  |  |  |  |
| rs73059359T>A | 127/136/37 | 240/283/77 | 0.36 | 0.724 | 0.93 (0.71-1.22) | 0.506 |
| rs7966327A>G | 100/151/49 | 218/294/88 | 0.39 | 0.549 | 1.11 (0.85-1.45) | 0.309 |
| *GLUT4* |  |  |  |  |  |  |
| rs5417C>A | 104/146/50 | 233/276/91 | 0.38 | 0.545 | 1.11 (0.85-1.44) | 0.303 |
| rs5415C>T | 154/116/30 | 315/232/53 | 0.28 | 0.268 | 1.07 (0.81-1.41) | 0.542 |
| rs5412G>A | 264/34/2 | 529/70/1 | 0.06 | 0.715 | 1.08 (0.63-1.86) | 0.706 |
| *GLUT5* |  |  |  |  |  |  |
| rs1063137G>A | 140/139/21 | 254/271/75 | 0.35 | 0.858 | 0.80 (0.60-1.06) | 0.039 |
| rs6658278G>A | 86/146/68 | 228/286/86 | 0.38 | 0.863 | 1.42 (1.09-1.84) | 0.001 |
| rs770041G>A | 290/9/1 | 537/59/4 | 0.06 | 0.105 | 0.33 (0.14-0.77) | 0.001 |
| rs12117043G>A | 143/134/23 | 240/280/80 | 0.37 | 0.930 | 0.74 (0.56-0.98) | 0.006 |
| rs3820034C>T | 209/85/6 | 410/168/22 | 0.18 | 0.399 | 0.91 (0.65-1.29) | 0.496 |
| rs34488100T>G | 191/101/8 | 381/196/23 | 0.20 | 0.800 | 0.96 (0.69-1.34) | 0.764 |
| rs1060998C>T | 197/94/9 | 331/237/32 | 0.25 | 0.233 | 0.66 (0.48-0.92) | 0.001 |
| *GLUT6* |  |  |  |  |  |  |
| rs3124757A>G | 236/59/5 | 461/129/10 | 0.12 | 0.710 | 0.91 (0.61-1.36) | 0.549 |
| *GLUT7* |  |  |  |  |  |  |
| rs12032857C>G | 266/34/0 | 478/114/8 | 0.11 | 0.671 | 0.49 (0.29-0.82) | 3.83×10^-4^ |
| rs10864379G>A | 189/100/11 | 419/169/12 | 0.16 | 0.364 | 1.37 (0.97-1.92) | 0.018 |
| rs72632913G>A | 221/75/4 | 418/172/10 | 0.16 | 0.128 | 0.84 (0.58-1.23) | 0.240 |
| rs34545462C>T | 266/34/0 | 534/64/2 | 0.06 | 1.000 | 0.98 (0.56-1.73) | 0.939 |
| *GLUT8* |  |  |  |  |  |  |
| rs3808835A>C | 207/89/4 | 458/136/6 | 0.12 | 0.342 | 1.42 (0.97-2.08) | 0.018 |
| rs5900747C>A | 120/146/34 | 263/274/63 | 0.33 | 0.522 | 1.11 (0.84-1.47) | 0.327 |
| *GLUT9* |  |  |  |  |  |  |
| rs10939650T>C | 81/155/64 | 171/289/140 | 0.47 | 0.413 | 0.99 (0.76-1.28) | 0.909 |
| rs6820230C>T | 232/63/5 | 509/86/5 | 0.08 | 0.573 | 1.59 (1.04-2.43) | 0.004 |
| rs3733589C>T | 122/143/35 | 236/280/84 | 0.37 | 0.931 | 0.93 (0.71-1.22) | 0.477 |
| rs4621429A>G | 206/87/7 | 479/114/7 | 0.11 | 0.833 | 1.72 (1.18-2.50) | 2.31×10^-4^ |
| rs3733591A>G | 131/129/40 | 266/262/72 | 0.34 | 0.584 | 1.05 (0.80-1.37) | 0.638 |
| *GLUT10* |  |  |  |  |  |  |
| rs707507C>T | 110/133/57 | 192/298/110 | 0.43 | 0.803 | 0.92 (0.71-1.20) | 0.429 |
| rs6094435C>T | 260/32/8 | 485/107/8 | 0.10 | 0.502 | 0.79 (0.51-1.22) | 0.158 |
| **Supplementary Table 6. Association of SNPs with lung cancer risk in the phase I case-control study (continue)** | | | | | | |
| **SNPs** | **Genotypes (WT/Het/Hom)^a^** | | **MAF** | ***P*_HWE_** | **adjust**  **OR (95% CI)^b^** | ***P* ^c^** |
|  | **Case (n=300)** | **Control (n=600)** |  |  |  |  |
| rs2235491G>A | 235/62/3 | 528/69/3 | 0.06 | 0.497 | 1.90 (1.20-3.01) | 3.60×10^-4^ |
| rs6017995A>G | 152/121/27 | 322/237/41 | 0.27 | 0.834 | 1.13 (0.85-1.51) | 0.267 |
| *GLUT11* |  |  |  |  |  |  |
| rs6003939A>C | 211/86/3 | 515/81/4 | 0.07 | 0.558 | 2.31 (1.51-3.53) | 3.49×10^-7^ |
| rs1129067C>T | 119/140/41 | 222/289/89 | 0.39 | 0.797 | 0.91 (0.69-1.19) | 0.345 |
| *GLUT12* |  |  |  |  |  |  |
| rs1484180G>A | 210/82/8 | 508/88/4 | 0.08 | 0.785 | 2.27 (1.51-3.40) | 1.85×10^-7^ |
| rs2200285G>A | 226/69/5 | 480/113/7 | 0.11 | 0.830 | 1.30 (0.87-1.93) | 0.093 |
| rs1385066T>C | 211/81/8 | 462/130/8 | 0.12 | 0.850 | 1.40 (0.97-2.02) | 0.019 |
| *GLUT14* |  |  |  |  |  |  |
| rs7309043G>A | 134/126/40 | 241/282/77 | 0.36 | 0.725 | 0.92 (0.70-1.21) | 0.438 |
| rs1894824C>T | 185/91/24 | 329/232/39 | 0.26 | 0.915 | 0.87 (0.64-1.17) | 0.213 |
| *HK1* |  |  |  |  |  |  |
| rs76042857G>A | 244/53/3 | 502/93/5 | 0.09 | 0.792 | 1.17 (0.75-1.83) | 0.353 |
| rs748235A>G | 146/119/35 | 273/256/71 | 0.33 | 0.358 | 0.92 (0.70-1.20) | 0.407 |
| rs1108272C>T | 77/155/68 | 188/297/115 | 0.44 | 0.934 | 1.20 (0.93-1.56) | 0.069 |
| rs1133189C>G | 222/73/5 | 435/150/15 | 0.15 | 0.631 | 0.90 (0.62-1.30) | 0.474 |
| rs10998710C>A | 108/147/45 | 161/299/140 | 0.48 | 1.000 | 0.71 (0.54-0.92) | 0.001 |
| *HK2* |  |  |  |  |  |  |
| rs2229627C>G | 96/146/58 | 158/320/122 | 0.47 | 0.101 | 0.87 (0.66-1.13) | 0.170 |
| rs10194657A>G | 86/146/68 | 141/319/140 | 0.50 | 0.142 | 0.89 (0.68-1.16) | 0.250 |
| rs2229626C>T | 110/141/49 | 208/282/110 | 0.42 | 0.402 | 0.93 (0.72-1.20) | 0.466 |
| rs656489G>A | 116/137/47 | 254/267/79 | 0.35 | 0.532 | 1.13 (0.87-1.47) | 0.232 |
| rs2229622T>C | 161/114/25 | 267/266/67 | 0.33 | 1.000 | 0.75 (0.56-1.00) | 0.009 |
| *HK3* |  |  |  |  |  |  |
| rs2278492C>T | 117/145/38 | 236/280/84 | 0.37 | 0.931 | 0.98 (0.74-1.28) | 0.813 |
| rs59411283C>T | 187/99/14 | 442/145/13 | 0.14 | 0.739 | 1.57 (1.12-2.20) | 0.001 |
| *PFKP* |  |  |  |  |  |  |
| rs1052333C>T | 134/119/47 | 238/282/80 | 0.37 | 0.861 | 0.94 (0.72-1.23) | 0.553 |
| rs541C>T | 208/84/8 | 376/199/25 | 0.21 | 0.902 | 0.76 (0.54-1.07) | 0.042 |
| rs1132173C>T | 85/142/73 | 189/296/115 | 0.44 | 1.000 | 1.19 (0.92-1.54) | 0.086 |
| rs10795005T>C | 164/112/24 | 312/248/40 | 0.27 | 0.356 | 0.96 (0.72-1.29) | 0.732 |
| rs4881086C>T | 223/71/6 | 392/181/27 | 0.20 | 0.300 | 0.66 (0.46-0.94) | 0.002 |
| rs10903966T>C | 63/155/82 | 173/306/121 | 0.46 | 0.565 | 1.35 (1.04-1.76) | 0.004 |
| rs4881106G>T | 164/105/31 | 305/244/51 | 0.29 | 0.842 | 0.95 (0.72-1.26) | 0.642 |
| rs10903967G>A | 187/98/15 | 379/189/32 | 0.21 | 0.219 | 1.01 (0.74-1.38) | 0.924 |
| rs2306302G>A | 239/56/5 | 499/93/8 | 0.09 | 0.135 | 1.20 (0.79-1.82) | 0.249 |
| rs4256893G>A | 78/140/82 | 161/301/138 | 0.48 | 0.935 | 1.12 (0.86-1.44) | 0.268 |
| rs11251720T>C | 263/37/0 | 471/120/9 | 0.12 | 0.687 | 0.51 (0.31-0.84) | 0.001 |
| rs3816704G>A | 244/55/1 | 513/85/2 | 0.07 | 0.763 | 1.36 (0.85-2.18) | 0.096 |
| **Supplementary Table 6. Association of SNPs with lung cancer risk in the phase I case-control study (continue)** | | | | | | |
| **SNPs** | **Genotypes (WT/Het/Hom)^a^** | | **MAF** | ***P*_HWE_** | **adjust**  **OR (95% CI)^b^** | ***P* ^c^** |
|  | **Case (n=300)** | **Control (n=600)** |  |  |  |  |
| rs35863365C>T | 231/64/5 | 465/129/6 | 0.12 | 0.552 | 1.04 (0.69-1.55) | 0.821 |
| rs79334726G>A | 204/88/8 | 472/122/6 | 0.11 | 0.682 | 1.72 (1.18-2.51) | 1.88×10^-4^ |
| rs34543032T>C | 248/49/3 | 532/67/1 | 0.06 | 0.713 | 1.66 (1.02-2.72) | 0.008 |
| rs4269839T>G | 231/63/6 | 468/122/10 | 0.12 | 0.555 | 1.05 (0.71-1.55) | 0.746 |
| *PFKL* |  |  |  |  |  |  |
| rs2847227T>C | 180/108/12 | 403/172/25 | 0.18 | 0.224 | 1.24 (0.90-1.70) | 0.083 |
| *PFKM* |  |  |  |  |  |  |
| rs11168418C>A | 161/110/29 | 333/229/38 | 0.25 | 0.915 | 1.13 (0.85-1.50) | 0.278 |
| rs1049392C>T | 237/56/7 | 419/163/18 | 0.17 | 0.658 | 0.66 (0.45-0.97) | 0.006 |
| *ALDOB* |  |  |  |  |  |  |
| rs4577C>T | 102/153/45 | 198/300/102 | 0.42 | 0.558 | 0.95 (0.72-1.24) | 0.594 |
| *ALDOC* |  |  |  |  |  |  |
| rs652677G>T | 209/81/10 | 386/185/29 | 0.20 | 0.257 | 0.81 (0.58-1.12) | 0.093 |
| *GAPDH* |  |  |  |  |  |  |
| rs6489721T>C | 123/135/42 | 173/306/121 | 0.46 | 0.565 | 0.67 (0.51-0.88) | 1.54×10^-4^ |
| rs7971637C>T | 163/111/26 | 262/266/72 | 0.34 | 0.717 | 0.72 (0.54-0.96) | 0.003 |
| *PGAM5* |  |  |  |  |  |  |
| rs11615090T>C | 228/69/3 | 456/132/12 | 0.13 | 0.472 | 0.97 (0.66-1.43) | 0.851 |
| rs12423651C>T | 60/153/87 | 172/301/127 | 0.46 | 0.870 | 1.41 (1.08-1.84) | 0.001 |
| rs4883606C>T | 200/92/8 | 356/213/31 | 0.23 | 1.000 | 0.73 (0.53-1.02) | 0.015 |
| rs117103262T>C | 272/25/3 | 541/57/2 | 0.05 | 0.660 | 1.01 (0.58-1.79) | 0.945 |
| *ENO1* |  |  |  |  |  |  |
| rs2274971A>G | 160/123/17 | 336/224/40 | 0.25 | 0.746 | 1.04 (0.77-1.40) | 0.726 |
| rs6660137A>G | 274/26/0 | 511/87/2 | 0.08 | 0.565 | 0.54 (0.30-0.99) | 0.009 |
| rs17032801A>G | 121/138/41 | 252/261/87 | 0.36 | 0.158 | 1.02 (0.78-1.32) | 0.874 |
| *ENO2* |  |  |  |  |  |  |
| rs11064467C>T | 159/112/29 | 408/175/17 | 0.17 | 0.887 | 1.85 (1.36-2.52) | 2.57×10^-7^ |
| rs2238116A>G | 185/102/13 | 363/196/41 | 0.23 | 0.051 | 0.88 (0.65-1.20) | 0.3 |
| rs710416A>C | 77/149/74 | 219/274/107 | 0.41 | 0.204 | 1.39 (1.08-1.80) | 0.001 |
| *ENO3* |  |  |  |  |  |  |
| rs112089924C>T | 187/109/4 | 402/182/16 | 0.18 | 0.486 | 1.12 (0.79-1.58) | 0.412 |
| rs238238A>G | 115/154/31 | 259/272/69 | 0.34 | 0.928 | 1.08 (0.82-1.42) | 0.477 |
| *ENO4* |  |  |  |  |  |  |
| rs9421246C>T | 88/155/57 | 213/288/99 | 0.40 | 0.932 | 1.22 (0.93-1.58) | 0.057 |
| rs11197835A>C | 242/53/5 | 480/118/2 | 0.10 | 0.071 | 1.03 (0.67-1.58) | 0.874 |
| rs2257791A>G | 122/146/32 | 304/256/40 | 0.28 | 0.188 | 1.41 (1.06-1.88) | 0.002 |
| *PKM* |  |  |  |  |  |  |
| rs12372964T>C | 240/56/4 | 454/140/6 | 0.13 | 0.265 | 0.81 (0.54-1.23) | 0.197 |
| *LDHA* |  |  |  |  |  |  |
| rs2249631G>A | 125/128/47 | 234/283/83 | 0.37 | 0.930 | 0.98 (0.75-1.27) | 0.813 |

| **Supplementary Table 6. Association of SNPs with lung cancer risk in the phase I case-control study (continue)** | | | | | | |
| --- | --- | --- | --- | --- | --- | --- |
| **SNPs** | **Genotypes (WT/Het/Hom)^a^** | | **MAF** | ***P*_HWE_** | **adjust**  **OR (95% CI)^b^** | ***P* ^c^** |
|  | **Case (n=300)** | **Control (n=600)** |  |  |  |  |
| rs7129689T>C | 100/132/68 | 194/300/106 | 0.43 | 0.617 | 1.08 (0.83-1.39) | 0.472 |
| *LDHC* |  |  |  |  |  |  |
| rs72868553C>A | 149/115/36 | 243/281/76 | 0.36 | 0.790 | 0.81 (0.62-1.07) | 0.049 |
| MAF, minimum allele frequency; HWE, hardy-weinberg equilibrium; OR, odds ratio; CI, confidence interval | | | | | | |
| ^a^ WT: wild type，Het: hybrid type，Hom: mutant homozygous type | | | | | | |
| ^b^ ORs were adjusted for age, sex, smoking status, drinking status, family history of tumor and lung cancer by the logistic regression model | | | | | | |
| ^c^ Taking α=0.01 as the significance level, Bonferroni was used to adjust the statistics with adjusted α '= 0.01/93= 1.08×10^-4^ | | | | | | |
